# Enhanced Positioning Algorithm Using a Single Image in an LCD-Camera System by Mesh Elements’ Recalculation and Angle Error Orientation

**DOI:** 10.3390/ma12244216

**Published:** 2019-12-16

**Authors:** Óscar de Francisco Ortiz, Manuel Estrems Amestoy, Horacio T. Sánchez Reinoso, Julio Carrero-Blanco Martínez-Hombre

**Affiliations:** 1 Department of Engineering and Applied Technologies, University Center of Defense, San Javier Air Force Base, MDE-UPCT, 30720 Santiago de la Ribera, Spain; 2 Mechanics, Materials and iManufacturing Engineering department, Technical University of Cartagena, 30202 Cartagena, Spain; manuel.estrems@upct.es (M.E.A.); horacio.sanchez@upct.es (H.T.S.R.); julio.carrero@upct.es (J.C.-B.M.-H.)

**Keywords:** image processing, position control, accuracy, micromachines, position compensation, inverse conical perspective, micromanufacturing, manufacturing systems, mechatronics

## Abstract

In this article, we present a method to position the tool in a micromachine system based on a camera-LCD screen positioning system that also provides information about angular deviations of the tool axis during its running. Both position and angular deviations are obtained by reducing a matrix of LEDs in the image to a single rectangle in the conical perspective that is treated by a photogrammetry method. This method computes the coordinates and orientation of the camera with respect to the fixed screen coordinate system. The used image consists of 5 × 5 lit LEDs, which are analyzed by the algorithm to determine a rectangle with known dimensions. The coordinates of the vertices of the rectangle in space are obtained by an inverse perspective computation from the image. The method presents a good approximation of the central point of the rectangle and provides the inclination of the workpiece with respect to the LCD screen reference system of coordinates. A test of the method is designed with the assistance of a Coordinate Measurement Machine (CMM) to check the accuracy of the positioning method. The performed test delivers a good accuracy in the position measurement of the designed method. A high dispersion in the angular deviation is detected, although the orientation of the inclination is appropriate in almost every case. This is due to the small values of the angles that makes the trigonometric function approximations very erratic. This method is a good starting point for the compensation of angular deviation in vision based micromachine tools, which is the principal source of errors in these operations and represents the main volume in the cost of machine elements’ parts.

## 1. Introduction

Positioning systems are increasingly present in all industrial processes. Furthermore, technology requires progressively more precise systems capable of positioning rapidly and robustly. The cost of those is one of the key factors to integrate high precision systems.

Thanks to the advances in screen and camera technology, positioning algorithms that analyze a pattern shown in a photographic image have been developed [1,2]. More recently, camera-screen positioning systems with dedicated artificial vision algorithms [3,4,5] have provided high precision at a very interesting cost compared to other positioning technologies such as encoders or resolvers.

Vision positioning systems are increasingly common in process automation [6,7,8,9,10], autonomous driving [11,12,13,14,15], or augmented reality assistants [16,17,18,19,20]. Indeed, this is one of the most promising elements in the Industry 4.0 revolution. However, the current positioning systems used in the machine tool industry based on high precision encoders and sensors are limited by their cost. Therefore, machine tools used for micro-manufacturing have very high prices and require large floor space. Due to this, in micro-manufacturing, the methods that use vision can be competitive by including high performance commercial elements and reducing space such as cameras and mobile phones’ LCD screens. In addition, such devices are increasing in definition and resolution, providing vision with much better accuracy.

The methodology used in this article to calculate the position and orientation of the camera in relation to the screen is based on pose determination [21,22,23,24], which is used to estimate the position and orientation of one calibrated camera. Several similar methods for calculating the position and orientation of a camera in space using a single image have been described and presented [22,25,26]. Nevertheless, pose estimation and marker detection are widely used tasks for many other technological applications such as autonomous robots [27,28,29], unmanned vehicles [30,31,32,33,34,35,36,37], and virtual assistants [38,39,40,41], among others.

Consequently, this article presents an enhanced method of recalculating the center of the image used by the positioning algorithm in an LCD-camera system, similar to that developed by de Francisco [4] and improved in subsequent studies [42], but being completely different from such previous studies regarding the procedure to calculate the positioning of the part with respect to the reference system of the screen. In previous works, the positioning was obtained through the global center of gravity of the 25 selected LEDs in the image. In this work, the position of the piece is calculated by previously determining an equivalent square obtained by means of regressions of the different lines that form the grid of the 25 LEDs.

In addition, this manuscript also presents the calculation and correction of the orientation angle, which, although very small, always influences the precision positioning due to the large distance between the location of the cutter and the screen. The new method is based on the calculation of the equivalent quadrangle that allows not only the positioning of the center of the image, but also the inclination. The method uses the treatment of an image to obtain the pixel coordinates of a 5 × 5 dot matrix that serves to locate the focus and orientation of the camera, where the error is due to the distance between the focus and the screen and can be assumed as sine error.

## 2. Materials and Methods

### 2.1. Experimental Setup and Measurements

**Figure 1:**
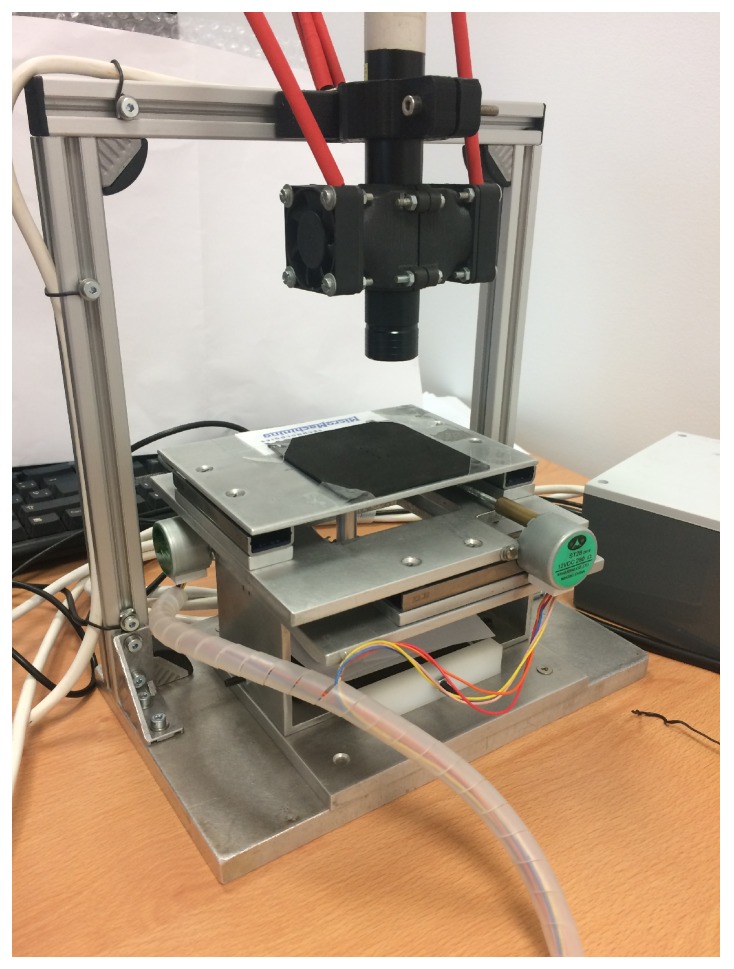
Model of the micromachine tool demonstrator used during the experimental test.

The experimental study was applied to a two-dimensional control system (*X* and *Y*). Figure 1 shows the model of the Micromachine Tool (MMT) demonstrator developed for this research. Two stepper motors (ST28, 12, 280 mA) controlled and moved two precision guides (IKO BSR2080 50 mm stroke), which were connected to a M3 ball screw/nut. The LCD screen used provided a 1136×640 pixel resolution, 326 ppi, and 0.078 mm dot pitch. The screen size was 88.5 × 49.9 mm. Both stepper motors were controlled by the digital output signals provided by an NI 6001-USB data acquisition card connected to the USB port of a laptop computer. The output signal of the acquisition card was treated by a pre-amplification power station composed of two L293 H-bridges. The control was programmed in LabVIEW. It received the image captured by the camera and processed it according to an image enhancing process. It consisted of an image mask application with color plane extraction, fuzzy pixel removal, small object removal, and particle analysis of the mass center of each evaluated pixel. Once it was processed using the developed artificial vision algorithm, it provided the positioning feedback signals needed to move the *X* and *Y* axes.

**Figure 2:**
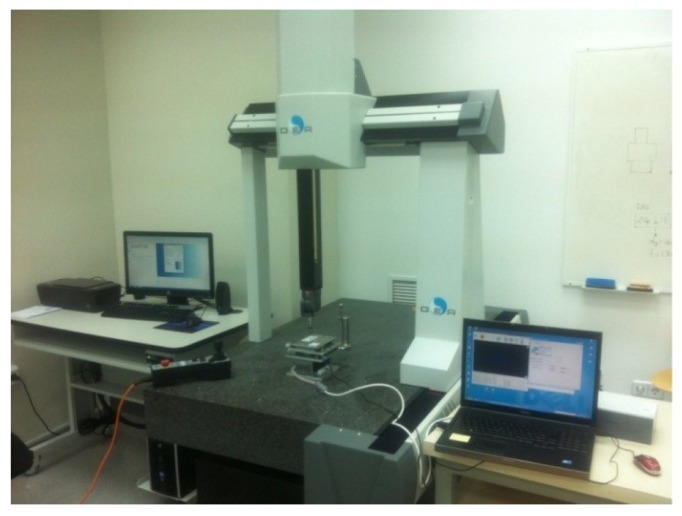
Setup used during the experimental test for the measurement with the Micromachine Tool (MMT) and the Coordinate Measuring Machine (CMM).

The images were taken by the camera included in the MMT, a Model MITSAI 1.3M digital camera with a resolution of 1280×1024 pixels (1.3 MPixels). To analyze the position, a Coordinate Measuring Machine (CMM) Pioneer DEA 03.10.06 with measuring strokes 600×1000×600 mm was used (Figure 2). The maximum permissible error of the DEA in the measurements was 2.8 + 4.0 L/1000 μm. The software used for the measurements was PC-DMIS.

Several tests were performed over a 2×2 gap pattern using the camera-LCD algorithm. The simulation consisted of testing a 5 mm *X* axis movement using 10 steps of 0.5 mm. Each travel was repeated 3 times in both the forward and backward direction, according to the the VDI/DGQ 3441 standard: Statistical Testing of the Operational and Positional Accuracy of Machine Tools - Basis.

### 2.2. Image Acquisition

Image acquisition was done using a procedure developed by the authors in VBA similar to that performed by software such as ImageJ© in its tool “Analyze Particles ...”.

The image may not be focused, although many webcams have autofocus mechanisms that make the focal length variable. In our case, it was unimportant because what matters was the bulk and its center of gravity. It should also be noted that if the extraction was from the complete image, the image usually contained the spherical errors of the lenses that focused the image onto the sensor.

In our case, to speed up the process and calculations, only the central area from the BMP image file that included all 25 LEDs was extracted. Only the red layer was analyzed because it was proven to be the most efficient and the only one used to generate the image. Given the size of the LEDs, an image size of 600×600 was sufficient to ensure the presence of at least 25 LEDs in the image.

## 3. Obtaining the Equivalent Quadrilateral

**Figure 3:**
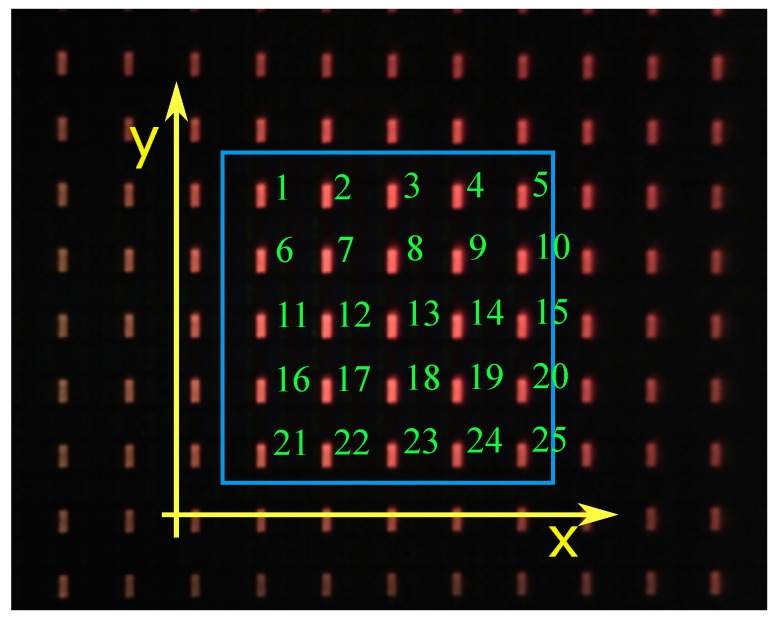
The 5 × 5 mesh captured by the camera with numbered elements.

Once the 25 coordinates of the centers of the LEDs were obtained, as seen in Figure 3, these data had to be statistically treated to obtain four vertices of a quadrilateral that collected information about the coordinates of the 25 points. With this quadrilateral and knowing the real side dimensions given by the size of the pixels, the position and orientation of the camera with respect to this square were obtained.

### 3.1. Regression of Lines

From the analysis of the 5 × 5 grid, different horizontal lines could be segregated, rearranging the table of coordinates by values in *y*, obtaining 5 groups of 5 values corresponding to the horizontal lines. Reordering by the values in *x*, the vertical lines were obtained in the same way. The 5 horizontal lines must be translated into 2 lines, the same with the vertical lines, so that the intersection of the four lines gave rise to the 4 vertices of the quadrilateral that represented a square in conical perspective. The two vanishing points were obtained by the intersection of opposite sides.

**Figure 4:**
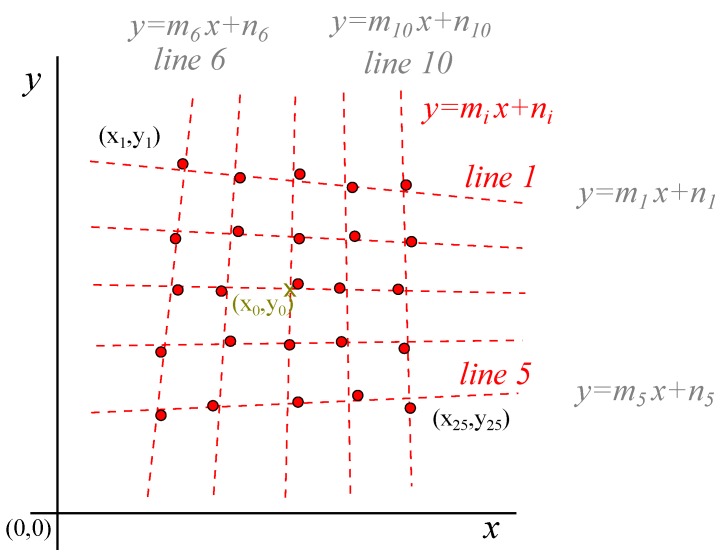
Regression lines in the 5 × 5 elements used in the image analysis.

**Figure 5:**
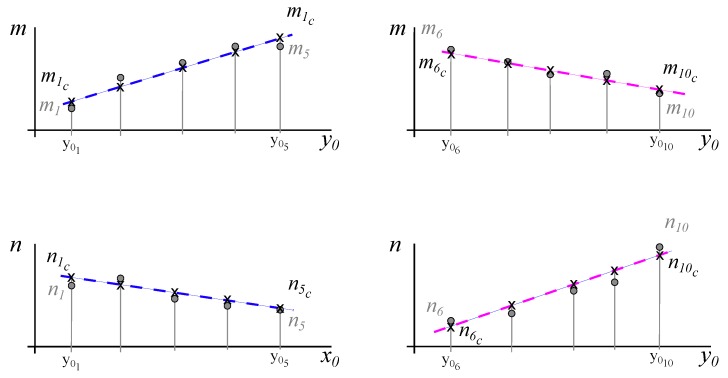
Regression lines (compensation) to optimize the position of the lines.

**Figure 6:**
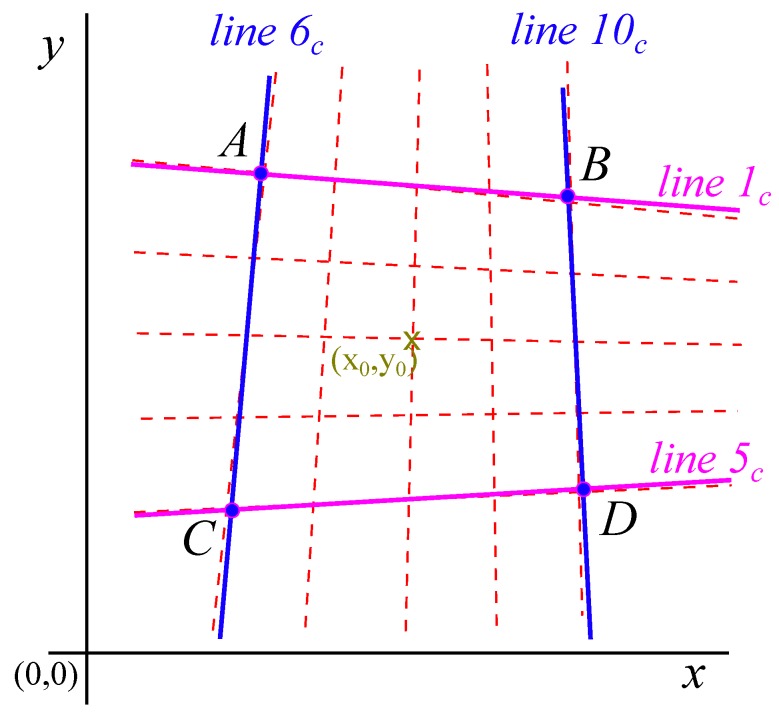
Final distribution of the rectangle used for the position and angle correction.

Figure 4 shows the regression lines, vertical and horizontal, that represented the different groups of points. The slope and interception terms of the lines followed a tendency that could be anyway also found as shown in Figure 5. These tendencies allowed the calculation of the different slopes in the extreme lines of the rectangle that represented adequately the 25 points, as the border of a chessboard included the dimension and position of the interior squares. The correlated lines and the rectangle used to determine the position and inclination of the axis of the camera are represented in Figure 6.

Since the angles of the slopes had very small variation, the line equations had the form $y=m_{i}x+n_{i}$. The intersection of a horizontal line with another vertical line is given by Equation (1):

(1) $$x=\frac{n_{j}+m_{j}n_{i}}{1-m_{i}m_{j}}$$

where subindex *i* corresponds to horizontal lines, while subindex *j* corresponds to vertical lines.

The steps to obtain the two horizontal lines were the following:

1. Sort by coordinate the data of the grid table obtained.
2. Separate this into groups of 5 points as they belong to the same line by similarity in coordinate *y*.
3. Perform regression of the five groups obtaining the equations of the five lines $y=m_{i}x+n_{i}$, i=1…5.
4. In a similar way, the data were sorted by *x* coordinates, then separated into 5 groups of 5 points, and the regression was performed obtaining the equations $y=m_{j}x+n_{j}$, j=6…10.
5. Obtain intersection points $\left(x_{i},y_{i}\right)$ from the central vertical line $y=m_{8}y+n_{8}$ with each of the horizontal lines.
6. Accomplish the regression of the slopes of the horizontal lines $m_{i}$ based on the vertical intersection coordinates $y_{i}$; thereby, the slope was obtained based on the vertical intersection.
7. In such a manner, we proceeded to select the slopes and the points through which the two horizontal lines indicated would be selected. The points of the extreme horizontal lines 1 and 5 were chosen. The two slopes of these two lines were calculated by means of the regression of the 5 slopes. The line was forced to pass through the intersection points of these extreme points, with the following remaining equations of the lines (Equations (2) and (3)):
  (2) $$y=m\left(y_{1}\right)x+\left(y_{1}-m\left(y_{1}\right)x_{1}\right)$$
  (3) $$y=m\left(y_{5}\right)x+\left(y_{5}-m\left(y_{1}\right)x_{5}\right)$$

A similar method was used for the calculation of the two vertical lines.

The intersection of the two almost parallel lines provided the 4 extreme vertices *A*, *B*, *C*, and *D* (Figure 6) that were introduced to the program of the inverse conical perspective to obtain the position and orientation of the camera in relation to the fixed coordinates located and oriented with the square that represented the grid of departure. The position and orientation of the contact point with respect to the screen reference system were obtained using an improvement of the method developed by Haralik for the rectangle reconstruction [22].

### 3.2. Example of the Calculation of Vertices

To obtain the straight lines, the slopes of the linear regression lines through the data point in the *x* and *y* arrays were calculated. In addition, the point at which a line intersected the *y* axis by using existing *x* and *y* values was also calculated for each vertex. The interception point was based on a best fit regression line drawn through the known *x* and *y* values, using an internal algorithm for the least squares regression procedure.

The starting point was the table of the centers of each of the zones sorted by the number of pixels comprising the area called mass (Table 1) as the number of pixels that included each zone.

Next, they were sorted by the *y* coordinates and were classified into groups that corresponded to the horizontal coordinates (Table 2).

As a result, the 5 horizontal lines were obtained $y=m_{i}x+n_{i}$, j=1…5 (Table 3).

In the same way, we proceeded to obtain the vertical lines $y=m_{j}+n_{j}$, j=6…10 (Table 4).

It is noted in Table 3 and Table 4 that all coefficients *m* and *n* had a tendency that could be also the object of a regression. This indicated that the lines were parallel as they had a vanishing point, and the plane that contained all the lines was not perpendicular to the focal line of the camera.

To find the two horizontal lines that represented the 5 lines, we proceeded to find the intersection points of the vertical center line with the 5 horizontal lines, obtaining the intersection points $\left(x_{i},y_{i}\right)$. The slope $m_{i}$ was correlated with the vertical coordinates $y_{i}$, obtaining the two slopes of the representative lines as lines that had a slope $m(y_{1})$ and $m(y_{5})$ and passing them through Points 1 and 5, respectively (Table 5).

Once having performed the regression of *m* with respect to $y_{i}$, a function of the slope that varied regularly across the different heights ($m=-1.136\times 10_{i}^{-3}+9.331\times 10^{-3}$) was obtained. As a result, the equations of the horizontal lines that passed through Points 1 and 5 could be calculated.

(4) $$y=-6108\times 10^{-3}x+540.807$$

(5) $$y=-9533\times 10^{-4}x+83.981$$

In the same way, we proceeded to obtain the 2 representative vertical lines:

(6) $$x=-6444\times 10^{-3}y+570.765$$

(7) $$x=-1277\times 10^{-3}y+112.547$$

The intersection of the opposite lines of this square provided the coordinates of the four vertices that represented the 25 LEDs (Table 6).

The vertices represented in Table 6 were treated using the photogrammetry method of the reconstruction of a rectangle described in Estrems [24], and the coordinates of the camera with respected to the square coordinate system were obtained, as well as the cosine direction of the focal line in this system.

(8) $$a=\sqrt{d_{f}^{2}-{\left(d_{f}\cdot cosb\right)}^{2}}=d_{f}\sqrt{1-cos^{2}b}$$

(9) $$a_{2}-a_{1}=d_{f}\left(\sqrt{1-cos^{2}b_{2}}-\sqrt{1-cos^{2}b_{1}}\right)$$

**Figure 7:**
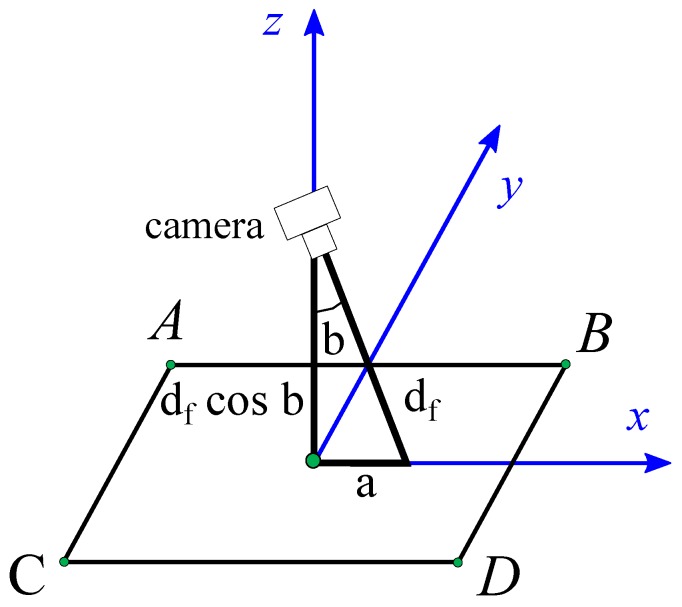
Position error in the vision system due to camera inclination for axis direction movement.

In Figure 7, the Abbe error *a* is represented and calculated by the focal distance $d_{f}$ and the cosine in the *z* direction cos *b*. The Abbe error is calculated in Equation (8), and the step error due to the variation of angle *b* during the movement is compensated at each point according to Equation (9).

## 4. Experimental Results and Discussion

The data obtained in the experimental test are described in Table 7, Table 8, Table 9, Table 10, Table 11 and Table 12, where CMM is the distance measure by the Coordinate Measuring Machine in the movements done by the MMT in every step during the test; Image is the distance moved in every step analyzed by the vision system; Error Image is the error provided by the vision system in every step after a comparison with the distance moved provided by the CMM; Compensation is the distance compensated due to angle error calculated in every step; Image compensated is the distance measured by the CMM after the application of the compensation calculated; Error compensation is the error after the compensation is applied; and Coincidence provides information about the coincidence in the orientation calculated for the angle compensation (YES means orientation coincidence, and NO means the angle orientation calculated is opposite the compensation required to minimize the error).

Table 13 summarizes the mean errors ($\bar{e}$) and the standard deviation errors (σ) calculated in each run of the experimental tests. The global mean (4.786 μm) and standard deviation (5.698 μm) were also calculated.

**Figure 8:**
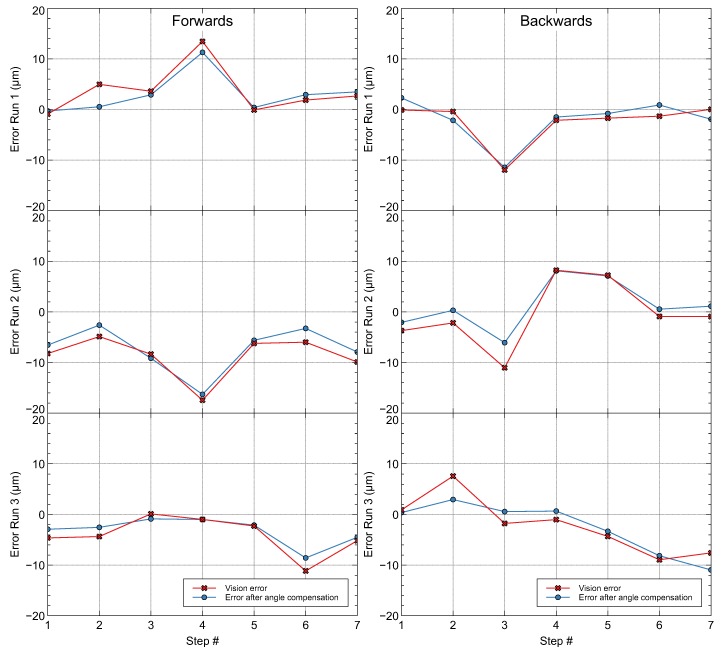
Error due to the vision system before and after angle compensation was applied.

As is seen in the graphs of Figure 8, the precision of positioning depended strongly on the initial error function, so the variation of the error was less than ±2μm, except several discrete points that were measured in a transition between columns of LEDs that were not so homogeneous in the LCD.

One remarkable result was that the orientation of the compensation error coincided with the sign of the error checked at almost all points. This indicated that there was variation of the orientation of the focal line with respect to the screen coordinate system, although this was not applied efficiently to improve the precision of measurement. This was probably due to the problems evaluating the trigonometric functions in angles with values less than $10^{-3}$ radians.

Therefore, due to the distance from the vanishing points of the lines, the estimation of the angular error did not turn out to be very precise in its quantification, although it provided qualitative descriptors on the direction and magnitude of the variation in the inclination of the camera.

## 5. Conclusions

A new method was developed to position the tool in a micromachine system based on a camera-LCD screen positioning system that also provided information on the angular deviations of the tool axis during operation.

The method gave a good approximation of the center point of the rectangle with a mean error of 0.96%, considering not only the vision algorithm, but also the mechanical test device, and provided the inclination of the workpiece with respect to the LCD-screen reference coordinate system.

The equivalent square was calculated as regression of the lines that could be drawn through the centers of gravity of each of the LEDs. The lack of parallelism between the sides of the square indicated an inclination of the camera axis relative to perpendicular to the screen. The variation of this inclination introduced errors in the displacements that were added to the simple displacement of the center of gravity and whose compensation was also calculated in this article.

A test of the method was designed with the assistance of a Coordinate Measurement Machine (CMM) to verify the accuracy of the positioning method. The test performed provided good accuracy in measuring the position of the designed method, but a high dispersion in the angular deviation was detected, although the orientation of the inclination was appropriate in almost all cases (85.7%). This was due to the small value of the angles that made the approximations of the trigonometric functions very erratic. With accurate formulas to approximate trigonometric functions for small angles, the method could help in obtaining more accurate measurements.

## Figures and Tables

**Table 1:** Table of the center of gravity example for Image 1.

Element #	*x*	*y*	Mass
1	112.591	195.242	1024
2	113.366	82.265	1015
3	226.495	196.041	990
4	112.105	309.020	982
5	227.189	83.003	968
6	226.096	309.625	961
7	340.272	197.033	947
8	340.892	83.472	943
9	112.080	423.237	938
10	567.863	198.706	925
11	453.996	197.528	917
12	454.825	84.746	897
13	339.734	310.306	890
14	568.349	86.025	873
15	225.773	423.599	872
16	453.313	311.012	857
17	567.139	311.749	854
18	111.869	536.665	833
19	339.310	423.985	813
20	566.355	425.027	786
21	225.561	537.419	776
22	452.760	424.705	757
23	339.075	537.371	717
24	452.296	537.806	682
25	565.838	538.361	673

**Table 2:** Groups of points corresponding to horizontal lines.

*x*	*y*
565.838	538.361
452.296	537.806
225.561	537.419
339.075	537.371
111.869	536.665
566.355	425.027
452.760	424.705
339.310	423.985
225.773	423.599
112.080	423.237
567.139	311.749
453.313	311.012
339.734	310.306
226.096	309.625
112.105	309.020
567.863	198.706
453.996	197.528
340.272	197.033
226.495	196.041
112.591	195.242
568.349	86.025
454.825	84.746
340.892	83.472
227.189	83.003
113.366	82.265

**Table 3:** Coefficients of horizontal lines.

*m*	*n*
3.331 × 10${}^{-3}$	536.395
4.127 × 10${}^{-3}$	422.710
6.018 × 10${}^{-3}$	308.298
7.393 × 10${}^{-3}$	194.394
8.142 × 10${}^{-3}$	81.126

**Table 4:** Coefficients vertical lines.

*m*	*n*
−5.774 × 10${}^{-3}$	568.910
−5.553 × 10${}^{-3}$	455.166
−4.050 × 10${}^{-3}$	341.114
−3.500 × 10${}^{-3}$	227.307
−3.080 × 10${}^{-3}$	113.355

**Table 5:** Intersection points of the central vertical line with horizontal lines.

Point	$x_{i}$	$y_{i}$
1	338.937	537.525
2	339.396	424.111
3	339.857	310.344
4	340.316	196.911
5	340.774	83.901

**Table 6:** Quadrilateral points to deal with the reverse perspective program.

Point	*x*	*y*
1	111.857	540.124
2	567.302	537.343
3	570.227	83.437
4	112.439	83.874

**Table 7:** Data for Run #1 forward with a mesh of 5 × 5 LEDs (values in μm).

CMM	Image	Error Image	Compensation	Image Compensated	Error Compensation	Coincidence
501	−501.897	−0.897	0.603	−501.294	−0.294	YES
1000	−995.031	4969	−4.432	−999.463	0.537	YES
1487	−1483.389	3611	−0.714	−1484.102	2.898	YES
1998	−1984.546	13.454	−2.164	−1986.710	11.290	YES
2489	−2489.081	−0.081	0.471	−2488.610	0.390	YES
2988	−2986.138	1.862	1.062	−2985.076	2.924	NO
3490	−3487.323	2.677	0.802	−3486.521	3.479	NO

**Table 8:** Data for Run #1 backward with a mesh of 5 × 5 LEDs (values in μm).

CMM	Image	Error Image	Compensation	Image Compensated	Error Compensation	Coincidence
496	−496.099	−0.099	2.394	−493.705	2.295	YES
992	−992.400	−0.400	−1.744	−994.144	−2.144	NO
1482	−1493.937	−11.937	0.505	−1493.432	−11.432	YES
1991	−1993.130	−2.130	0.632	−1992.498	−1.498	YES
2494	−2495.707	−1.707	0.912	−2494.795	−0.795	YES
2993	−2994.327	−1.327	2.214	−2992.113	0.887	YES
3494	−3493.969	0.031	−1.950	−3495.918	−1.918	YES

**Table 9:** Data for Run #2 forward with a mesh of 5 × 5 LEDs (values in μm).

CMM	Image	Error Image	Compensation	Image Compensated	Error Compensation	Coincidence
490	−498.217	−8.217	1.671	−496.545	−6.545	YES
985	−989.868	−4.868	2.260	−987.608	−2.608	YES
1481	−1489.347	−8.347	−0.803	−1490.150	−9.150	NO
1976	−1993.442	−17.442	1.153	−1992.290	−16.290	YES
2493	−2499.212	−6.212	0.604	−2498.608	−5.608	YES
2991	−2996.982	−5.982	2.728	−2994.254	−3.254	YES
3485	−3494.890	−9.890	1.978	−3492.912	−7.912	YES

**Table 10:** Data for Run #2 backward with a mesh of 5 × 5 LEDs (values in μm).

CMM	Image	Error Image	Compensation	Image Compensated	Error Compensation	Coincidence
495	−498.695	−3.695	1.631	−497.065	−2.065	YES
991	−993.169	−2.169	2.490	−990.679	0.321	YES
1486	−1497.023	−11.023	4.948	−1492.075	−6.075	YES
2003	−1994.767	8.233	−0.105	−1994.872	8.128	YES
2501	−2493.756	7.244	−0.142	−2493.898	7.102	YES
2995	−2995.898	−0.898	1.446	−2994.452	0.548	YES
3494	−3494.915	−0.915	2.048	−3492.867	1.133	YES

**Table 11:** Data for Run #3 forward with a mesh of 5 × 5 LEDs (values in μm).

CMM	Image	Error Image	Compensation	Image Compensated	Error Compensation	Coincidence
496	−500.617	−4.617	1.720	−498.897	−2.897	YES
994	−998.341	−4.341	1.814	−996.527	−2.527	YES
1497	−1496.865	0.135	−0.990	−1497.855	−0.855	YES
1997	−1997.961	−0.961	−0.007	−1997.968	−0.968	NO
2500	−2502.260	−2.260	0.168	−2502.092	−2.092	YES
2996	−3007.135	−11.135	2.567	−3004.567	−8.567	YES
3498	−3503.156	−5.156	0.660	−3502.496	−4.496	YES

**Table 12:** Data for Run #3 backward with a mesh of 5 × 5 LEDs (values in μm).

CMM	Image	Error Image	Compensation	Image Compensated	Error Compensation	Coincidence
498	−497.074	0.926	−0.532	−497.606	0.394	YES
999	−991.402	7.598	−4.628	−996.030	2.970	YES
1492	−1493.747	−1.747	2.333	−1491.414	0.586	YES
1992	−1992.997	−0.997	1.684	−1991.313	0.687	YES
2487	−2491.310	−4.310	1.007	−2490.303	−3.303	YES
2985	−2993.930	−8.930	0.791	−2993.139	−8.139	YES
3486	−3493.567	−7.567	−3.367	−3496.934	−10.934	NO

**Table 13:** Summary of the errors, in absolute value, provided by the proposed vision positioning algorithm.

	#1 Forward	#1 Backward	#2 Forward	#2 Backward	#3 Forward	#3 Backward	Global
Mean (μm)	3.936	2.519	8.708	4.883	4.086	4.582	4.786
Mean (%)	0.79	0.50	1.74	0.98	0.8	0.92	0.96
σ (μm)	4.771	4.238	4.212	6.586	3.699	5.567	5.698
σ (%)	0.95	0.85	0.84	1.32	0.74	1.11	1.19

## Abbreviations

The following abbreviations are used in this manuscript:

LCD	Liquid Crystal Display
MMT	Micromachine Tool
ppi	points per inch
CMM	Coordinates Measuring Machine
